# Early Host Interactions That Drive the Dysregulated Response in Sepsis

**DOI:** 10.3389/fimmu.2019.01748

**Published:** 2019-08-06

**Authors:** Steven W. Kerrigan, Tatyana Devine, Glenn Fitzpatrick, Jecko Thachil, Dermot Cox

**Affiliations:** ^1^Cardiovascular Infection Research Group, Royal College of Surgeons in Ireland, Dublin, Ireland; ^2^School of Pharmacy, Royal College of Surgeons in Ireland, Dublin, Ireland; ^3^Molecular and Cellular Therapeutics, Royal College of Surgeons in Ireland, Dublin, Ireland; ^4^Irish Centre for Vascular Biology, Royal College of Surgeons in Ireland, Dublin, Ireland; ^5^Department of Haematology, Central Manchester University Hospitals NHS Foundation Trust, Manchester, United Kingdom

**Keywords:** sepsis, endothelial cell, platelets, hyper-activation, micro-organisms

## Abstract

Sepsis is defined as life-threatening organ dysfunction caused by a dysregulated host response to infection. While many individual cells and systems in the body are involved in driving the excessive and sometimes sustained host response, pathogen engagement with endothelial cells and platelets early in sepsis progression, are believed to be key. Significant progress has been made in establishing key molecular interactions between platelets and pathogens and endothelial cells and pathogens. This review will explore the growing number of compensatory connections between bacteria and viruses with platelets and endothelial cells and how a better understanding of these interactions are informing the field of potential novel ways to treat the dysregulated host response during sepsis.

## Introduction

**Figure 1:**
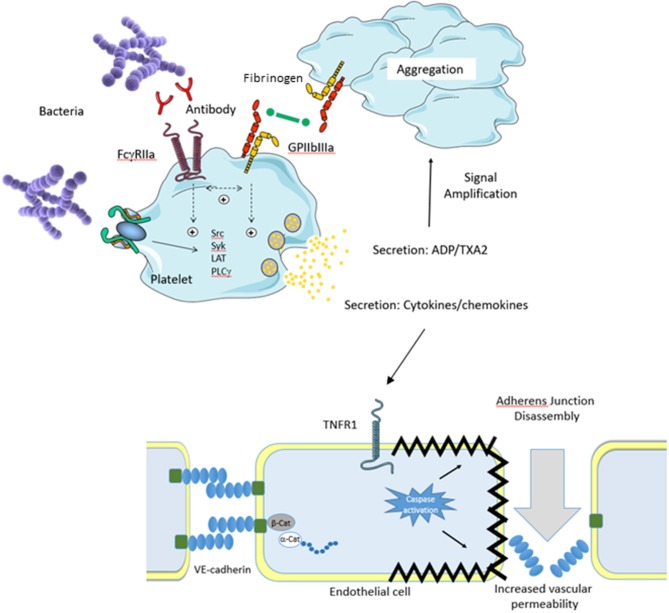
Platelet Theory. An invading pathogen binds to platelets either directly (absence of plasma protein) or indirectly (presence of plasma protein). Binding results in platelet activation via Src kinases which results in prostanoid release, cytokine secretion, granule secretion, and activation of GPIIbIIIa. Release of ADP and thromboxane A2 (TXA2) serves to amplify the platelet response. In conjunction with this, activation of GPIIbIIIa allows fibrinogen binding resulting in platelet aggregation. Secretion of platelet cytokines and chemokines activates the vascular endothelium. For example, secreted TNFa activates the TNFR1 receptor on endothelial cells which triggers the death pathway resulting in apoptosis. This results in endothelial cell shrinkage and loss of barrier integrity leading to increased vascular permeability and shock. Separation of endothelial cells allows for pathogens to escape the bloodstream and infect major organs which eventually leads to multi organ failure.

**Figure 2:**
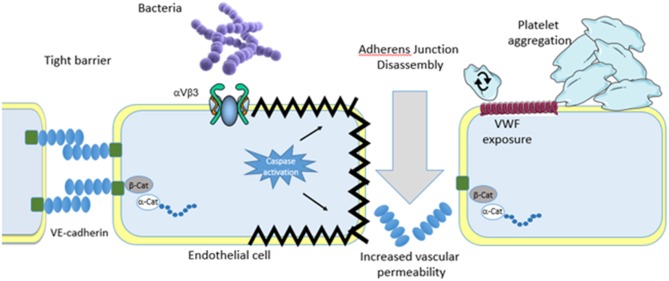
Endothelial theory. Similar to platelets invading pathogens bind either directly or indirectly to the vascular endothelium. Binding results in endothelial cell activation that results in apoptosis. Although the pathway to apoptosis has not yet been defined apoptosis leads to endothelial cell shrinkage and loss of barrier integrity leading to increased vascular permeability and shock. Separation of endothelial cells allows for pathogens to escape the bloodstream and infect major organs which eventually leads to multi organ failure. Endothelial cell activation also leads to granule secretion which deposits vonwillebrand factor on the surface of the endothelial cells. This encourages platelet rolling, activation and aggregate formation. In addition, bacteria also roll on the deposited vonwillebrand factor eventually coming to a halt and firmly adhering. Passing platelets bind to the immobilized bacteria on the endothelial cells which leads to inappropriate thrombus formation and exacerbation of the dysregulation.

Sepsis is defined as life-threatening organ dysfunction caused by a dysregulated host response to infection (Sepsis 3) and despite being the primary cause of in-hospital mortality there is little in the drug discovery pipeline for this disease (1). Treatment primarily focuses on the use of antibiotics but with the growing incidence of antibiotic-resistant strains of bacteria and the time it takes to diagnose sepsis there is clearly a need to discover novel approaches to treating sepsis. As the definition indicates that sepsis is a dysregulated host response (2) an obvious novel treatment strategy is to correct this dysregulated host response. Through significant advances in our understanding of the molecular interactions two possible theories have emerged that help explain the nature of the dysregulation. The platelet-pathogen theory suggests that pathogens bind to platelets activating them. These activated platelets bind to both endothelial cells and immune cells activating them which causes damage and disruption to the endothelial layer, leading to loss of barrier integrity, fluid leakage resulting in shock (Figure 1). Alternatively, the endothelial-pathogen theory suggests that pathogens bind to endothelial cells activating them. This leads to a release of granules and pro-inflammatory cytokines and chemokines, that recruit platelets to form a thrombus encasing the pathogens and immune cells and contribute to excessive thrombocytopenia and hyper-inflammatory response. Pathogen binding to endothelial cells also causes apoptosis which results in disruption of the endothelial layer leading to adherens junction disassembly, increased vascular permeability, fluid leakage, and shock (Figure 2). In this review, we will discuss each of these theories outlining the molecular mechanisms leading to each.

## Platelet Function

After red blood cells platelets are the most numerous cell-like particle in the blood. Their total volume and surface area when combined together is larger than that of all the leukocytes taken together. They are not true cells as they have no nuclei and are in fact fragments of megakaryocytes (3). Their primary role is in hemostasis and they clump together to occlude any breach in the vasculature. They can be considered to have three distinct functions: adhesion, secretion and aggregation and platelet activation links these processes through multiple signaling pathways.

### Adhesion

Platelets typically travel close to the endothelium patrolling for breaches in the vasculature. The resting endothelium is inert ensuring platelets do not adhere, however when the endothelial cell layer is damaged exposing sub-endothelial matrix proteins (which as occurs with a cut), or becomes activated through inflammation the area becomes highly thrombogenic and platelets subsequently adhere. Key proteins in the matrix that support platelet adhesion are collagen and von Willebrand Factor (VWF). Adhesion is a highly controlled event and is mediated by platelet receptors such as integrin α2β1 and glycoprotein (GP) VI or collagen and GPIb/IX/V for VWF. The VWF-GPIb/IX/V interaction is shear-dependent and is primarily involved in adhesion under high shear stress such as in coronary arteries. See Nieswandt et al. for a review on platelet function (4).

### Activation

While binding to either collagen or VWF facilitates adhesion of platelets to the site of injury it also triggers activation of the platelet leading to platelet secretion and aggregation. The adhesion process is not the only process for activating platelets as soluble mediators can also lead to platelet activation. Substances like adenosine diphosphate (ADP), thrombin, thromboxane A2, and adrenaline can also trigger platelet activation through their respective receptors. To further link all of the platelet functions the primary source of these soluble mediators are platelets in a process known as secretion. Most of these receptors mediate their effects through two different signaling pathways. Some receptors stimulate phospholipase (PL) A2 which ultimately leads to the production of thromboxane A2 while others trigger PLC activation (5).

### Secretion

Once activated, platelets secrete the contents of their granules. Platelets contain different types of granules such as alpha and dense granules and lysosomes. The content of these granules plays an important role in haemostasis. Secreted ADP activates the surrounding platelets and this is critical in creating the growing clot. The granules also secrete adhesion molecules such as fibrinogen as well as up-regulating adhesion receptors such as GPIIb/IIIa (fibrinogen receptor) and p-selectin (CD62) to the platelet surface (6).

### Aggregation

While platelet activation leads to granule secretion it also leads to activation of GPIIb/IIIa on the platelet surface. GPIIb/IIIa is an integrin (αIIbβ3) fibrinogen receptor and is usually present in a resting, non-binding conformation. Once activated it can bind soluble fibrinogen and as fibrinogen is a large bivalent molecule one fibrinogen molecule can bind to two GPIIb/IIIa molecules. If these GPIIb/IIIa molecules are on different platelets the effect is to link two platelets together. Considering that there are around 50,000 GPIIb/IIIa molecules per platelet this creates a platelet-rich clot cross-linked by fibrinogen. This process is known as aggregation and is critical for sealing a breach in the vasculature (7).

## Platelet Signaling

Platelet activation occurs through two processes—outside-in signaling and inside-out signaling and is reviewed in more detail by Stalker et al. (8). The classic example of outside-in signaling is the process by which soluble mediators such as ADP, thrombin, and thromboxane (Tx) A2 activate platelets. These bind to membrane receptors (in most cases G-protein-coupled receptors) triggering downstream events. Agonist binding to these receptors trigger activation of one of two phospholipases (PL)—PLA2 or PLC. PLA2 is the primary PL and acts to release arachidonic acid from the inner membrane of the platelet. This arachidonic acid is a substrate for cyclooxygenase (COX). In platelets, the COX isoform is COX 1 and it converts arachidonic acid into prostaglandin (PG) H2. PGH2 is an intermediate in the signaling process and it is further metabolized to its active product by cell specific enzymes. In platelets the primary enzyme is thromboxane synthase which converts PGH2 to TxA2 which binds to receptors and triggers further platelet activation. This pathway is completely inhibited by COX 1 inhibitors such as aspirin.

The second signaling pathway uses a number of different isoforms of PLC which cleave inositol triphosphate (IP3) and diacylglycerol (DAG) from the membrane. IP3 binds to an intracellular Ca2+ channel which increases intracellular Ca2+ levels through release from the endoplasmic reticulum. DAG ultimately activates protein kinase C which activates further downstream events. This pathway is not inhibited by aspirin. Agonists are often divided into weak agonists which are PLA2-dependent and strong agonists which are PLC-dependent, although this is often concentration-dependent with low concentrations of agonists using PLA2 and high concentrations using PLC.

While soluble agonists activate platelets via PLA2/PLC this is not the only process for activating platelets. Platelet adhesion also leads to platelet activation however, the activation process for each of the adhesion receptors is receptor specific. One example is that of platelet activation in response to adhesion to fibrinogen. The platelet receptor involved is GPIIb/IIIa and initially it was considered that GPIIb/IIIa was merely an adhesion receptor as there were no obvious signaling pathways associated with it. However, it is now clear that GPIIb/IIIa can recruit signaling molecules [Src family kinases, focal adhesion kinase (FAK) etc.] and generate activating signals (9). GPIb/IX/V acts by recruiting 14-3-3ζ, actin binding protein, Src, FAK etc (10). Receptors that contain an ITAM (immunoreceptor, tyrosine-based activation motif) or ITAM-like domain such as Fc receptors and CLEC-2 recruit the tyrosine kinase syk when dimerized (11). Fc receptors can heterodimerise, that is they can dimerize with other receptors such as GPVI and GPIb. GPVI contains an SH3 domain that recruits the src family kinases and when dimerized with FcR-γ it triggers the recruitment of syk and phosphorylation of FcR (12).

The wave of outside-in activation is followed by a wave of inside-out signaling. This primarily involves talin binding to the β3 subunit of GPIIb/IIIa (13). This inside-out signaling is essential for full activation of the platelet.

Platelets also have inhibitory signaling pathways to counter the activating pathways. The primary pathway is mediated by prostacyclin (PGI2). When it binds to is receptor it increases cAMP which in turn activates PKA and inhibits platelet activation. A related mechanism is that of nitric oxide (NO) which directly enters the platelet and activates soluble guanylate cyclase increasing cGMP levels. Both prostacyclin and NO are produced by healthy endothelial cells to prevent clot formation.

In sepsis, thrombocytopenia develops in up to 50% of cases and is associated with poor outcome (14). This thrombocytopenia is likely to play a significant role in the pathogenesis of sepsis leading to development of multiple organ dysfunction syndrome (MOPS), disseminated intravascular coagulation (DIC) and/or massive bleeding as a result of platelet consumption and thrombus formation (15). It is well-established that innate immune cells (IIC) such as macrophages, natural killer cells (NK) cells, neutrophils, dendritic cells etc, release a plethora of pro-inflammatory mediators creating a so-called cytokine storm (16). We now also know that in addition to their hemostatic functions platelets also play a role in inflammation and regulation of inflammatory response by secreting cytokines, interferons, and chemokines. For example, *Staphylococcus* and *Streptococcus* spps can trigger platelet aggregation, cytokine release, and thrombocytopenia (17–19).

## Platelets and Immunity

The critical role of platelets in the innate immune response is largely mediated by their ability to interact with other immune cells mainly neutrophils (20, 21). Platelets express receptors on their surface that are usually associated with immune cells such as FcγRIIa and Toll-like receptors (TLR) 2 and 4 (22). For example, in 2007 Clark et al., demonstrated that lipopolysaccharide (LPS) binds to platelet TLR4 which mediates attachment to neutrophils. Critical to this interaction is platelet activation which results in granule secretion, P-selectin expression on the surface of the platelet, and crosslinking to its counter receptor P-selectin glycoprotein ligand-1 on the leukocyte surface. Other studies have demonstrated that platelet GPIbα can bind VWF and crosslink the platelet to neutrophils via the β2 integrin (CD18). Neutrophil Extracellular Traps (NETs) are web-like structures composed of a chromatin backbone, histones and anti-microbial proteins and their main function is to trap and kill bacteria, virus, and fungi, avoiding their dissemination. While NET formation is a critical event in innate immunity, uncontrolled formation may exert significant tissue damage which contributes significantly to the already difficult to control host dysregulation (23). Dengue virus has been shown to activate platelets in a CLEC-2-dependent manner producing extracellular vesicles that induce NET formation (24). Regardless of the interaction, platelet attachment to the neutrophil results in rapid activation and most importantly the formation of NETs and together they play an important role in the pathogenesis of sepsis (25).

As platelet activation occurs during inflammation and infections such as sepsis there is also a need to control excessive platelet activation. One controlling factor is that of C-reactive protein (CRP) which is an acute phase protein synthesized in the liver in response to infection. It exists in a monomeric (mCRP) and pentameric (pCRP) forms which have opposing effects. pCRP is known to inhibit platelet aggregation by binding to GPIIb/IIIa and thus will act to reduce thrombus formation (26). Another agent that regulates the platelet response is nitric oxide (NO). NO is produced by the endothelium and is a potent vasodilator but also an inhibitor of platelet activation. During sepsis NO levels increase due to production by immune cells. This increased NO contributes to vasodilation and hypotension as well as inhibiting platelet function (27, 28). Thus, platelet activation status during sepsis depends on the balance between activating and inhibiting factors.

### Platelet Immune Receptors

Platelets express immunoreceptor tyrosine-based activation motifs (ITAMs)-containing receptors such as FcγRIIa, GPVI, and C-type lectin-like receptor (CLEC)-2 (29). The presence of FcγRIIa, a receptor for the Fc portion of IgG, on platelets is unusual as it is a receptor involved in phagocytosis and all other FcγRIIa-expressing cells are phagocytic (29). However, while not true phagocytic cells platelets do engulf bacteria in a manner that has some similarities to phagocytosis (30, 31). Platelet FcγRIIa is fully functional and can trigger platelet aggregation. Immune complexes [or even heat-agglutinated immunoglobulin (Ig) G] directly induce platelet aggregation in an FcγRIIa-dependent manner. Furthermore, bacteria that become coated in IgG can also induce platelet aggregation in an FcγRIIa- dependent manner (see below). The functionality of TLRs is more complex. While studies show that TLRs can mediate platelet activation others show that they don't (32). There is evidence to suggest that platelets activated by TLRs can engage with neutrophils and/or monocytes triggering their activation (33). Dendritic Cell-Specific Intercellular adhesion molecule-3-Grabbing Non-integrin (DC-SIGN), also known as CD209, is a C-type lectin which is usually expressed on macrophages and dendritic cells that is known to be involved in the phagocytosis of HIV and is also expressed on platelets (34). DC-SIGN and FcγRIIa are particularly implicated in platelet activation in Dengue virus infection (DENV) and incubation of platelets with anti-DC-SIGN antibodies prevented DENV-mediated platelet activation (35).

Other receptors shown to facilitate platelet interactions in response to pathogens are sialic-acid-binding immunoglobulin-like lectins (Siglecs)- a type I transmembrane proteins, that play role in regulating the host's immune responses to pathogen (36). In platelets Siglec-7 is most abundantly expressed and its function depends on the P2Y1 platelet receptor and of the GPIIb/IIIa integrin. It is proposed that Siglec-7 down-regulates pathogen-induced platelet activation by inducing apoptosis (37). Along with FcγRIIa, CLEC-2, and GPVI are ITAM receptors found on platelets. The ligands for CLEC-2 is podoplanin and for GPVI it is collagen, fibrinogen, and fibrin (38). These receptors have been found to play a role in the interaction with pathogens. Thus, CLEC-2 has been shown to bind to human immunodeficiency virus (HIV) and GPVI has been shown to bind to Hepatitis C virus (39, 40). GPVI has been shown to be important in *Klebsiella pneumoniae* sepsis models (41). CLEC-2 has been shown to drive thrombosis following Salmonella infection (42). Furthermore, the platelet CLEC-2-podoplanin interaction has been found to be an important modulator of inflammation during sepsis (43, 44).

### Platelet Cytokines

Platelets release cytokines either directly into the bloodstream by de-granulation, or by secreting platelet-derived micro-vesicles (PDMV), which make up between 60 and 90% of extracellular vesicles (EV) in plasma and contribute to hemostatic and immune function of platelets (45–47). These “immuno-parcels” can elicit innate and adaptive immune responses at distant sites by delivering variety of immunomodulatory factors, such as CD154 (also known as soluble CD40 Ligand, sCD40). CD154 from PDMV is enough to activate antigen specific splenic B cell response in CD154^−/−^ mice, in both T cell-dependent and independent manner (48). PDMV also contain a variety of nucleic acids including messenger and micro-RNAs (49). For example, platelets contain mRNA of pro-IL-1β, which upon platelet activation is translated *in situ* and fully synthesized pro-IL-1β is then released into circulation (50). IL-1β but not IL-1α binds to fibrinogen and it is the bound form of IL-1β that has enhanced action to induce monocyte chemoattractant protein 1 (MCP-1) and nitric oxide (NO) production by endothelial cells via NFκB pathway (51). Among other pro-inflammatory modulators released by platelets are: MCP-1, macrophage inflammatory protein (MIP)-1α, regulated on activation, normal T cell expressed and secreted (RANTES), IL-8, tumor growth factor (TGF)-β, angiogenesis and growth factors, and various immunoglobulins (48, 52–55).

## Platelet Bacterial Interactions

There are several platelet receptors that are involved in either direct interactions with pathogens either through direct interactions between microbial adhesins and platelet surface component or indirect associations via a bridging molecule (17, 20, 56, 57). The best studied interaction is that between *S. aureus* and platelets but the interaction with other Gram-positive and Gram-negative bacteria has also been described (17). The most significant causative agents of sepsis are *S. aureus* is the major cause (21%), *E. coli* (16%), *Staphylococcus epidermidis* (11%), and *S. pneumoniae* (4%) and these are also the best studied for their interactions with platelets (58).

### *Staphylococci*-Platelet Interactions

*S. aureus* expresses several cell wall anchored surface proteins that enable binding of the bacteria to platelets (59). During the exponential growth phase *S. aureus* expresses Clumping factor (Clf) B, fibronectin-binding protein (FnBP) A and B; while ClfA is expressed during the stationary phase. These proteins bind to fibrinogen facilitating its binding to and activation of platelet GPIIb/IIIa (60–62). *S. aureus* surface protein A (SpA) is known to bind IgG but also binds to domain A1 on VWF which mediates binding to platelet GPIbα (63). However, these interactions are insufficient to activate platelets and all require simultaneous activation of FcγRIIa through engagement with opsonising IgG's on the surface of the bacterium. Engagement of integrins and FcγRIIa lead to platelet activation, aggregation, adenosine triphosphate (ATP) release, and thrombus formation. In the absence of plasma proteins *S. aureus* can also bind directly to GPIIb/IIIa via its iron-regulated surface determinant protein B (IsdB) inducing platelet adherence and aggregation (64). Four more platelet activating proteins that are secreted by *S. aureus* have been recently identified by Binsker et al: Extracellular adherence protein (Eap), the chemotaxis inhibitory protein of *S. aureus* (CHIPS), the formyl peptide receptor-like 1 inhibitory protein (FLIPr), and the major autolysin Atl (AltA) which were all shown to induce P-selecting expression, while Eap, CHIPS, and AltA also induced platelet aggregation (65). *Staphylococcus epidermidis* expresses serine–aspartate repeat protein (Sdr) G, a member of Microbial Surface Components Recognizing Adhesive Matrix Molecules (MSCRAMM), that engage with GPIIb/IIIa either via fibrinogen or directly (66).

### *Streptococcci*-Platelet Interactions

A large number of streptococci have been shown to interact with platelets including *Streptococcus gordonii, Streptococcus sanguinis, Streptococcus oralis, Streptococcus agalactiae*, and *Streptococcus pneumoniae* (17). A common mechanism through which the streptococci interact with platelets is via a serine-rich repeat protein (Srrp). This Srrp is expressed on the surface of many streptococci (67–70). Numerous studies have demonstrated that the Srrp binds to platelet GPIbα and induces activation. Under low shear conditions (<500 s^−1^) platelets roll along immobilized streptococci using a mechanism characteristic of the interaction observed when platelets interact with VWF. Deletion of the Srrp ablates the interaction with platelets under both static and shear conditions (68, 70). *S. gordonii* have also been shown to bind GPIIb/IIIa. A common feature in this interaction is the presence of an RGD-like sequence in a large cell wall surface protein (3,500 amino acid residues) that mediates the binding called Platelet Adherence protein A (PadA) (71, 72). Binding resulted in src induced rearrangement of the platelet actin cytoskeleton leading to filopodia and lamellipodia formation resulting in platelet spreading over the bacteria (73). Protein analysis and site directed mutagenesis revealed that PadA contains two critical integrin-recognition motifs (383RGT and 484AGD) that mediate interaction with GPIIb/IIIa (73). *S. pneumoniae* has also been shown to bind to and induce platelet activation resulting in dense granule secretion in a TLR2-dependent manner. Although the bacterial component that interacts with platelet TLR2 was not identified a likely candidate is wall lipoteichoic acid (74).

### Platelet-Gram-Negative Bacteria Interactions

*Escherichia coli, Helicobacter pylori, Porphyromonas gingivalis*, and *Brucella abortus* all have been shown to bind platelets (18, 75–78). *E. coli* O157:H7 interact with platelets via platelet TLR4 and P-selectin (CD62) leading to secretion of CD40L, increase in fibrinogen binding on platelets and the formation of aggregates (79). This platelet activation is both FcγRIIa- and GPIIb/IIIa-dependent, and requires opsonisation of bacteria with IgG (18, 32). Platelet activation induced by *H. pylori* has been shown to be FcγRIIa and GPIbα-dependent (77, 80). *B. abortus* binds directly to platelets in a dose-dependent manner, although platelet receptors for this interaction remain to be established. This interaction induced enhanced fibrinogen binding and P-selectin expression, and promoted infection of monocytes by delivering bacteria to them (75). *P. gingivalis* has also been shown to bind to platelets in an IgG-dependent manner. Depletion of IgG or pre-incubation of platelets with an anti-FcγRIIa antibody abolished platelet activation and aggregation (81).

### Complement-Dependent Platelet Activation

While some bacteria have surface proteins that can interact with platelets many bacteria can activate platelets despite the absence of such proteins. *S. aureus* mutants which lack known platelet interacting proteins such as ClfA and ClfB and are thus unable to bind fibrinogen can still induce platelet activation. This is also true of strains of *S. sanguinis* that do not bind to platelets. However, the aggregation profile of these bacteria is quite different. While wildtype *S. aureus* induces aggregation within 2–3 min these non-interacting bacteria take more than 15 min to induce aggregation although the aggregation is still FcγRIIa-dependent. Bacterial-induced aggregation is usually mediated by FcγRIIa and a co-receptor such as GPIIb/IIIa and GPIb, in the case of non-binding bacteria the co-receptor is a complement receptor. This slow aggregation requires complement assembly most likely by the alternative pathway. The delay in onset of aggregation probably reflects the time required for complement membrane attack complex formation (82, 83).

## Platelet-Virus Interactions

**Figure 3:**
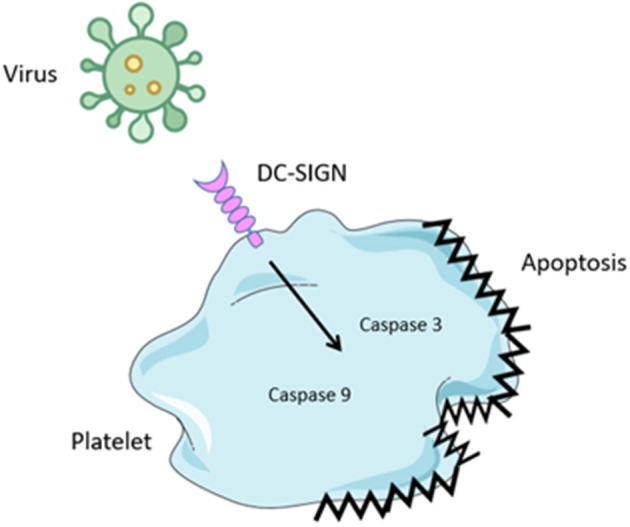
Virus binding to platelets. Several virus have been shown to bind to platelets. Binding is mediated by attachment to the Dendritic Cell-Specific Intercellular adhesion molecule-3-Grabbing Non-integrin (DC-SIGN), a c-type lectin receptor. Engagement results in activation of caspase 3 and 9 leading to platelet apoptosis. This results in thrombocytopenia.

Bacteria are not the only pathogens that affect platelet function during infection. Viral Haemorrhagic Fevers (VHF's) are very contagious zoonotic diseases that occur all over the world although more prevalent in tropical and warm climates (84). As name suggests VHF are associated with thrombocytopenia, hemorrhage, and fever caused by systemic inflammation. VHF viruses cause diseases such as Ebola, Lassa, Marburg, Yellow fever, and Dengue (69). Viruses interact with platelets mainly via FcγRIIa, integrins, DC-SIGN, and complement receptors (85). The best characterized of these interactions is with the Dengue virus which binds to DC-SIGN on platelets, causing their activation, mitochondrial dysfunction, and apoptosis via caspase-9 and 3 engagement thus contributing to systemic inflammation and platelet depletion (Figure 3) (35).

## Endothelial Cell Function

Endothelial cells make up a highly adaptive single cell layer displaying distinct apical and basolateral sides in blood vessels. They appear elongated in the direction of blood flow and form a tight cobblestone pattern. Endothelial cells are highly metabolically active and are constantly sensing alterations in the local extracellular environment (86). The endothelium receives and integrates information from hormones, neurotransmitters, pericytes, smooth muscle cells, leukocytes, platelets, viral or bacterial infection, proinflammatory cytokines, and oxygen tension (87). Endothelial cells also respond to vascular injury and high hydrodynamic shear stress. The primary function of the endothelium is to regulate systemic blood flow and maintain blood vessel wall permeability which selectively controls the movement of fluid, ions, and macromolecules between the circulating blood and the surrounding tissues.

### Barrier Integrity

The passage of molecules, cells, and fluid through the endothelial cell layer is a tightly controlled process. In the healthy state endothelial cells are held together to ensure barrier integrity through intracellular junctions called tight junctions and adherens junctions (87). Critical tight junction proteins include occludins and claudins which are localized at the apical area of the intracellular cleft. These tight junction proteins are predominantly responsible for control of permeability of solutes between blood and tissues (88). Adherens junction proteins on the other hand are predominantly responsible for maintaining endothelial cell-endothelial cell connection to ensure vascular integrity. The main adherens junction protein responsible for this is VE-cadherin which is localized at the basal membrane (89, 90).

### Anticoagulant and Anti-thrombotic Surface

Under resting conditions the luminal surface of the endothelium is typically both anticoagulant and antithrombotic in order to maintain fluidity within the circulation (91). Healthy endothelial cells express a number of inhibitors to prevent the synthesis and activity of the key penultimate enzyme in the coagulation cascade, thrombin. Anti-coagulation is ensured when thrombin interacts with the endothelial cell integral membrane protein thrombomodulin. Engagement induces activation of protein C which forms a complex with protein S and results in inactivation of factor VIIIa and factor Va, critical co-factors for coagulation (92). Sustained or prolonged release of mediators from healthy endothelial cells inhibit activation and adhesion of platelets to the endothelium. Prostacyclin (PGI2) is released from resting endothelial cells and binds to the Gs-coupled prostacyclin I2 receptor (IP receptor) expressed on platelets (93). Receptor activation results in activation of protein kinase A which increases platelet cAMP, thus preventing platelet activation. Similarly, lipid-soluble nitric oxide is also released from resting endothelial cells and activates protein kinase G which increases cGMP, also preventing platelet activation. Increases in cAMP or cGMP inhibit platelet aggregation, platelet secretion and platelet adherence to the vessel wall (94).

### Leukocyte Recruitment

The endothelium responds to tissue invasion by transporting leukocytes from the bloodstream to subendothelial compartments. Circulating leukocytes i.e., neutrophils and monocytes are recruited to the source of infection and release TNFα, a potent endothelial cell activator. TNFα induces the expression of adhesion molecules on the surface of endothelial cells facilitating leukocyte rolling-and-adhesion. MLK is involved in the release of Weibel-Palade bodies (WPBs) stored in the endoplasmic reticulum which harbor P-selectin (95). P-selectin is subsequently transported, by WPBs, and presented on the luminal side of the endothelium (96). The initial interaction between endothelium and leukocyte involves selectins (P- and E-selectins). These selectins recognize sialyl-Lewis-x moieties of leukocyte glycoproteins allowing reversible adherence to the endothelium (97). A second interaction between lymphocyte function-associated antigen 1 (LFA-1) and macrophage-1 antigen (MAC-1) on the surface of leukocytes with intracellular adhesion molecule (ICAM)-1 and ICAM-2 on the endothelium occurs, however, the initial interaction is weak which allows leukocytes to roll along the vasculature (98, 99). Chemokines, such as CXCL8 upregulated by NF-κB and AP-1, bind to receptors on leukocytes inducing a conformational change in LFA-1 and MAC-1 (CD18/CD11b) allowing them to adhere to ligands with high affinity (100). This facilitates firm adhesion of leukocytes to the endothelium arresting rolling movement. In a process known as extravasation, LFA-1, MAC-1 (CD18/CD11b), and platelet endothelial cell adhesion molecule (PECAM, CD31) enable the leukocytes to squeeze between endothelial cell junctions. The leukocytes migrate to the basement membrane (diapedesis) where enzymes break down the extracellular matrix (101). The leukocytes continue migrating through subendothelial tissue following a chemokine (CXCL8; CCL2) concentration gradient, a process known as chemotaxis, to the source of infection where they encounter pathogens.

## Endothelial Cell-Bacterial Interactions

### Gram Positive Bacteria-Endothelial Cell Interactions

Bacterial interaction with endothelial cells is not well-defined and as a result few interactions have been identified. Lack of progression in this field can be attributed to poor models used to study the interactions. Endothelial cells exist in a dynamic environment surrounded by various circulatory cells (leukocytes, red blood cells, platelets) in plasma. To better reflect the physiological conditions during infection Cheung and Fischetti demonstrated that when endothelial cells are grown in the presence of tumor necrosis factor α (TNFα), significantly more *S. aureus* bind to the endothelial cells. *S. aureus* binding was further increased when fibrinogen was added to the endothelial cells (102). These findings suggest that *S. aureus* binds to fibrinogen and cross-links the bacteria to the “activated” endothelial cell (103). Binding was abolished when *S. aureus* cells were treated with trypsin, suggesting that the bacterial adhesin was a cell wall protein. Using surface-biotinylated solubilized components of *S. aureus* revealed a critical role for protein A in binding endothelial cells (104). Protein A is known to bind a number of plasma proteins including IgG and VWF (63, 105). Subsequent studies demonstrated that upon endothelial cell activation, release of intracellular calcium causes mobilization of weibel palade bodies which results in deposition of VWF onto the surface of the cell, thus producing a binding site for protein A on *S. aureus*. Claes et al. identified a second VWF binding protein expressed on S. aureus called vwb (106). This protein typically acts as a coagulase and activates prothrombin to generate fibrin. Much similar to before deposition of VWF on the surface of endothelial cells following activation or injury provides a binding site for vwb. A *S. aureus* strain deficient in the vwb protein or an antibody against the A1 domain of vWf significantly reduced *S. aureus* adhesion to endothelial cells an *in vivo* model of blood stream infection. Collectively these data suggest that *S. aureus* use at least two different mechanisms to interact with surface deposited VWF after endothelial cell activation. While these observations are critical in our understanding of how *S. aureus* interacts with the endothelium it doesn't identify the primary interaction that triggers endothelial cell activation to lead to VWF deposition. Using a shear based model, McDonnell et al., identified a very early interaction that drives vascular dysregulation early in infection (107). Using primary human endothelial cells sheared at physiological shear rates experienced in the vasculature the authors demonstrated that the *S. aureus* ClfA binds plasma fibrinogen and crosslinks the bacteria to the major endothelial cell receptor αVβ3. Binding via this mechanism resulted in VWF deposition on the surface of the endothelial cells which will allow both bacterial and platelet attachment. Binding also resulted in a loss of barrier integrity as determined by an increase in vascular permeability and loss of VE-cadherin expression. Permeability changes is a common characteristic in sepsis patients and facilitates dissemination of infection to all major organs, thus contributing to organ failure. Blocking *S. aureus* attachment to αVβ3 prevented VWF deposition and loss of barrier integrity. S. aureus attachment also triggered significant cytokine and chemokine release contributing to hyper-inflammation and immune cell recruitment (108).

While it is evident that *S. aureus* has evolved to possess various mechanisms to attach to endothelial cells the functional significance of these interactions are still under investigation. One clear functional interaction demonstrates the ability of *S. aureus* to internalize into endothelial cells (109). Internalization likely occurs to evade immune or anti-microbial attack, as neither immune cells or antibiotics are capable of entering into endothelial cells. Internalization is mediated by Fnbp expressed on *S. aureus* which binds plasma fibronectin and cross links to endothelial cell receptor α5β1.

### Gram Negative Bacteria-Endothelial Cell Interactions

In contrast to *S. aureus* interactions with endothelial cells which focused primarily on identifying bacteria proteins, research investigating the interaction between Gram negative bacteria interaction and endothelial cells has focused on its major cell wall component LPS and the downstream signaling as a result of this interaction. Possibly the best described interaction involves the Toll-Like Receptors (TLRs). For example, TLR4 recognizes LPS (110). TLR4 signaling begins with the formation of a TLR4/myeloid differentiation 2 (MD2) complex. Upon LPS binding, homodimerization of two TLR4/MD2 receptors occurs, inducing a conformational change that allows the Toll/interleukin-1 receptor-like (TIR) domains of TLR4 to recruit adaptor proteins for the activation of MyD88-dependent pathway at the plasma membrane. These adaptor proteins subsequently activate interleukin (IL)-1R associated kinases (IRAKs) and tumor necrosis factor (TNF) receptor associated factor 6 (TRAF6) (111, 112). This, in turn, activates transforming growth factor β-activated kinase 1 (TAK1) resulting in MAP kinase kinase (MKK) inducement of the MAPK signaling cascade (113). The MAPK signaling cascade activates nuclear transcription factors such as nuclear factor (NF)-κB and activator protein (AP)-1 (114). The activation of NF-κB and AP-1 induces the production of pro-inflammatory cytokines and chemokines driving the acute phase inflammatory response (115). Furthermore, LPS can stimulate a MyD88-independent pathway following internalization of the TLR4-MD2 complex (116). TLR4-MD2 complex utilizes adaptor proteins TIR domain-containing adaptor inducing IFN-β (TRIF), TIR domain-containing adaptor molecule-1 (TICAM-1), and TRIF-related adaptor molecule (TRAM) to activate TNF receptor associated factor 3 (TRAF3) (117–119). TRAF3 activates the kinase TBK1 and IKKε stimulating interferon regulatory factor 3 (IRF3) nuclear translocation, resulting in the production of type-I interferons (116). Type-I interferons are associated with upregulation of anti-inflammatory cytokines IL-10 and IL-27 which inhibit acute phase pro-inflammatory cytokine (TNFα and IL-1) and chemokine (CXCL1 and CXCL2) production (120–122). Nuclear upregulation of both pro- and anti-inflammatory genes corresponds to type-II endothelial activation.

While there is little doubt that LPS plays a key role in driving the inflammatory response during sepsis Gram negative bacteria i.e., *E. coli* can also bind to endothelial cells. McHale et al., demonstrated that the highly conserved outer membrane protein A (ompA) binds directly to aVb3 on endothelial cells in the absence of plasma proteins (123). In this unique interaction, the ompA protein contains the RGD integrin recognition motif that binds directly to the RGD binding site on αVβ3. Similar to *S. aureus* binding to αVβ3, *E. coli* attachment results in loss of barrier integrity causing an increase in permeability and loss of VE-cadherin expression.

## Endothelial Cell-Virus Interactions

**Figure 4:**
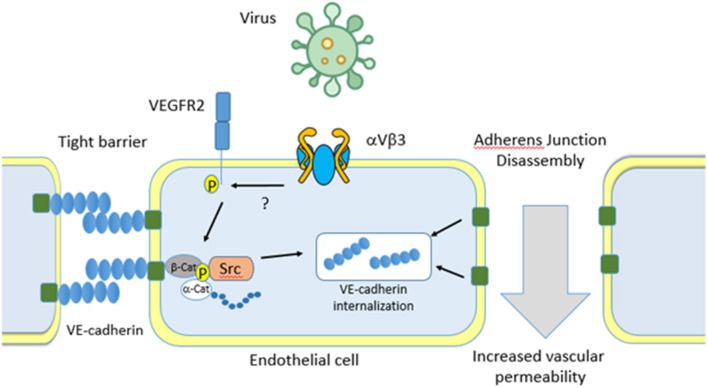
Virus binding to endothelial cells. Virus can bind to the major endothelial cell receptor aVb3. Binding results in recruitment of the Vascular Endothelial cell Growth Factor Receptor 2 (VEGFR2) which activates Src kinase. This directs VE-cadherin internalization, dissociation of adherens junctions, and an increase in vascular permeability. Separation of endothelial cells allows for pathogens to escape the bloodstream and infect major organs which eventually leads to multi organ failure.

While it generally accepted that virus are capable of binding to and dysregulating the endothelial cell barrier, the mechanisms through which they interact is not well-characterized. For example, the dengue virus envelope protein has been shown to bind to host cell Fc receptors, DC-SIGN (CD209), ICAM3 (CD-50), CD14, mannose receptor (CD206), HSP70/90, GRP78, and heparan sulfate proteoglycans (HSPGs), all of which are expressed on endothelial cells (124–129). In addition, hantaviruses have been shown to bind to endothelial cell αVβ3 which recruits VEGF receptor 2 to activate Src mediated internalization of VE-cadherin. Internalization causes loss of barrier integrity resulting in localized increases in vascular permeability and oedema (Figure 4) (130–134).

## Novel Targets in Sepsis

As our basic understanding of the molecular mechanisms through which bacteria interact with either platelets or endothelial cells develops, key novel targets that drive dysregulation in both of these cells is becoming clear. While it can be argued that both platelets and endothelial cells may be innocent by-standers in these diseases there is strong evidence to support a role for them in driving the early signals that tips sepsis into a state of excessive and sustained host dysregulation. For example, if, as we have discussed above, platelets respond to pathogens by becoming activated they will aggregate forming micro-thrombi. These micro-thrombi can occlude the micro-circulation in many organs such as liver, kidney and brain. These occlusions cause ischemic damage which, as it accumulates, leads to organ failure. Furthermore, serotonin released from these activated platelets causes severe vasodilation leading to shock (135). The combination of organ damage and shock is what defines sepsis. On the other hand, several studies have demonstrated that upon entry to the bloodstream, bacteria bind to the vascular endothelium within minutes. Bacterial binding causes endothelial cell injury that results in loss of barrier integrity which causes the down-regulation of the critical adherens junction protein VE cadherin. This process facilitates bacterial dissemination to all major organs causing secondary infection and therefore contributing to organ failure. In addition, bacteria binding to the endothelium results in a significant cytokine and chemokine release driving the hyper-inflammatory response during sepsis.

As a result of our improved understanding of the molecular interactions that drive dysregulation in the bloodstream, it has led to identification of key novel targets that could control sepsis better. For example, a number of meta-analysis of retrospective studies showed that patients on aspirin or clopidogrel who develop sepsis have better outcomes than those not on an anti-platelet agent (136). This is despite the fact that patients on an anti-platelet agent are generally much older and sicker (usually anti-platelet agents are used post-MI) than those not on an anti-platelet agent. As conventional anti-platelet agents are designed to inhibit the hemostatic properties of platelets what about a new generation of anti-platelet agent that inhibits the interaction of the platelet with the pathogen? For instance a preliminary study has shown benefit of targeting the CLEC-2 ligand podoplanin in mouse models of acute respiratory distress syndrome (44).

There are three approaches to targeting platelets in sepsis. The first is to target platelet activation. This can easily be achieved using existing anti-platelet agents—primarily aspirin and clopidogrel. The advantage is that there is a lot of experience with these agents and both are off patent and thus inexpensive. This is particularly important in treating sepsis in developing countries. There is good evidence to suggest that these agents have benefit in sepsis from retrospective studies and certainly a good quality prospective study is warranted (137, 138). The disadvantage with these agents is the potential for bleeding complications. This is especially true for patients with thrombocytopenia where there is a risk of preserving platelet number at the expense of platelet function. However, the meta-analysis suggests that this risk is more than compensated by the benefit. One factor with the meta-analyses is that patients were already on anti-platelet therapy prior to developing sepsis. This is the ideal situation as it prevents platelets being activated. However, when a patient is diagnosed with sepsis and then given aspirin it might be a bigger challenge as thrombocytopenia will already be established. Thus, they need to be given anti-platelet agents as early as possible to maximize their benefit.

A precision medicine approach can also be used where the platelet receptor that binds to the pathogen is targeted. For instance, GPIIb/IIIa is the receptor for *S. aureus* and thus GPIIb/IIIa antagonists have the potential to prevent platelet activation in *S. aureus*-mediate sepsis (139). However, the difficulty here is that the pathogen must be identified prior to treatment. The big challenge in sepsis is identification of the pathogen. Once identified antibiotic therapy is the only effective solution. Another difficulty is that many of these receptors are involved in haemostasis and thus their inhibition will lead to increased bleeding problems.

A third approach is to target platelet-immune receptors. This has the potential to prevent platelet activation induced by pathogens without compromising their hemostatic properties. Furthermore, they are pathogen-independent—or at least involved with many pathogens. One example of such a strategy is to target FcγRIIa on the basis that most bacteria use it as a co-receptor for platelet activation. Small molecules that inhibit FcγRIIa have been discovered and a monoclonal antibody against FcγRIIa is entering PI studies (140, 141). Such an agent could be given to patients prior to confirmation of sepsis. This would slow the progression of sepsis allowing time for appropriate antibiotic therapy to take effect. Not only would this improve survival it may reduce the incidence and severity of post-sepsis syndrome. Furthermore, as it does not impact haemostasis there is no risk of bleeding with patients and the identity of the pathogen is not necessary.

Similarly, given the unique and critical finding that a growing number of pathogens bind directly to the vascular endothelium using the same receptor, αVβ3, inhibition of this receptor may prevent endothelial injury thus preventing the patient from progressing to shock. In addition, by preventing pathogens from internalizing into endothelial cells may also help reduce the incidence of recurrent infection which is common in sepsis, a step is also partly mediated by αVβ3. Currently there are no drugs available to prevent bacterial attachment to the vascular endothelium and therefore endothelial cell dysregulation is difficult to control, however identification of the molecular interactions between bacteria and the endothelial cells makes it an attractive future target.

## Clinical Implications—Perspectives

As described before, platelet activation is a key factor in the pathogenesis of sepsis, but what has been crucially lacking in this regard are (i) widespread acceptance and acknowledgment of the fundamental role of platelets in this area, (ii) the understanding that platelet activation can lead to microthrombi from platelet aggregation which can then lead to single or multiple organ failure, (iii) thrombocytopenia in sepsis is at least partly (or predominantly in authors' opinion) due to platelet aggregation in addition to decreased platelet production and destruction by the micro-organisms, and (iv) inadequacy of the trials using antiplatelet agents in conjunction with the standard therapies in the comprehensive management of sepsis. One of the fundamental issues in translational research in this area has been delineating when the platelet activation is protective in the fight against the infections from the destructive role by forming platelet aggregates and microthrombi. Clarification of the timing when the beneficial role changes to a damaging role can aid in targeting the antiplatelet therapy before organ failure has developed. In addition, identifying which specific receptors and molecular mechanisms are involved in the different infections and at different stages would help in selecting appropriate antiplatelet therapies rather than using the conventional antiplatelet drugs in all cases.

An additional area of interest is dealing with the platelet-endothelial interactions and how they may be perturbed in sepsis (142). Although there have been many studies on the topic in the setting of cardiovascular diseases, it is still early days for clinical interventions in sepsis. Two key experimental trials have shown promise by inhibiting histones and neutrophil extracellular trap formation, which are key players in the platelet-endothelial interactions. Esmon's group showed in an animal model of sepsis that they can protect the host from DIC by specifically blocking the protein, histone H4 (143). A more recent paper noted the formation of cell-free DNA and NETS in sepsis (144). They also correlated with sepsis severity. Importantly, the use of recombinant DNAse could cause the degradation of NETs which could attenuate organ damage in combination with antibiotics.

## Author Contributions

All authors listed have made a substantial, direct and intellectual contribution to the work, and approved it for publication.

### Conflict of Interest Statement

The authors declare that the research was conducted in the absence of any commercial or financial relationships that could be construed as a potential conflict of interest.
